# Minimally Invasive Osteosynthesis of Distal Tibia Fractures using Anterolateral Locking Plate

**DOI:** 10.5704/MOJ.1811.008

**Published:** 2018-11

**Authors:** P Choudhari, D Padia

**Affiliations:** Department of Orthopaedics, Sri Aurobindo Medical College and Post Graduate Institute, Indore, India

**Keywords:** tibia, anterolateral, fracture, plate

## Abstract

**Introduction:** Plating in distal tibia fractures are associated with higher rate of soft tissue complications. As adequate soft tissue cover is available over anterolateral surface of the tibia, use of anterolateral plate fixation in distal tibia fractures has increased. The purpose of our research is to evaluate the outcomes of anterolateral locking plate fixation in distal tibia fractures using ORIF.

**Materials and Methods:** A retrospective analysis of 25 patients, who had distal tibia fractures and underwent open reduction and anterolateral plating. Bone and soft tissue healing and complications encountered were analysed.

**Result:** Full weight bearing was allowed at an average of 5.4 months (range: 3-12 months) after seeing radiological union. We have observed superficial wound infection in four cases. Two cases had marginal necrosis, two cases had sensory disturbance over dorsolateral aspect of foot and two cases had delayed non-union. Mean length of surgical incision was 9cm (range: 5-12 cm).

**Conclusion:** Open reduction internal fixation of distal tibia fractures with anterolateral plating is a reliable way of fracture fixation and stabilisation with proper surgical technique and aseptic precautions.

## Introduction

Right from conservative to surgical management using techniques such as external fixators, intra medullary nailing and internal fixation have been used in the management of distal tibia fractures^1-6^. There has been no agreement over the superiority of any one method over the other in this type of fractures as all the methods of surgical procedures have their own pros and cons^4^. One also has to emphasise on soft tissue healing as well in these types of fractures for a favourable outcome^7-11^.

Open reduction and internal fixation (ORIF) using plate do cause soft tissue trauma but it also helps to achieve a good fracture reduction which eventually leads to proper healing of the fractures^12-13^, provided an optimum soft tissue handling has been done. Many studies have shown reasonable results with minimally invasive osteosynthesis of distal tibia fractures using anterolateral tibia plating but has many complications such as non-union and malunion^14-18^. There also have been certain studies that have shown poor results with ORIF with anterolateral plating^19-23^. Moreover, the results will depend on severity of injury, soft tissue trauma, surgical timing, surgical techniques and comorbid illnesses of the patient^5, 6, 24^. Patients with and without fibular fracture fixation along with distal tibia fractures were included in distal tibia fracture studies.

The purpose of our research is to evaluate the outcomes of anterolateral locking plate fixation in distal tibia fractures using ORIF.

## Materials and Methods

**Fig. 1:**
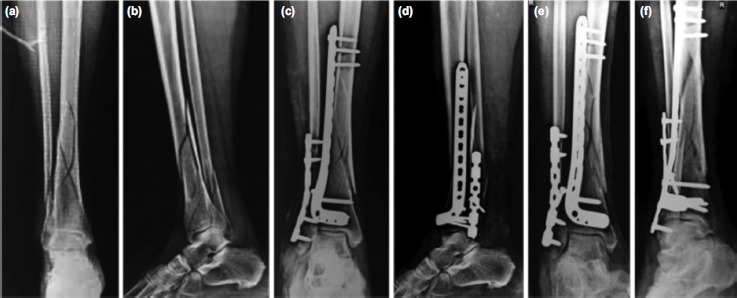
Pre-operative radiographs of intra-articular distal tibia-fibula fracture AO Type 44-C3 showing (a) antero-posterior and (b) lateral view. While, immediate post-operative radiographs showing ORIF with plating done in (c) antero-posterior view and (d) lateral view. Three months follow-up radiographs of ORIF with plating showing (c) antero-posterior and (d) lateral view.

**Fig. 2:**
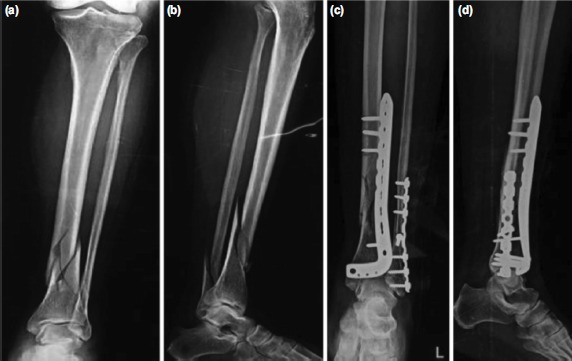
Pre-operative radiographs of extra-articular distal tibia fracture AO Type 43-B3 showing (a) antero-posterior and (b) lateral view. Immediate post radiographs showing ORIF with plating done in (c) antero-posterior and (d) lateral view.

Twenty-five patients with distal tibia fractures were retrospectively analysed from 2014 to 2016 out of which 20 were males and 5 were females with mean age of 41.5 (range: 25-75 years). Fractures were classified according to AO classification. Open fractures were not included in this study. After subsidence of the swelling all the fractures were fixed in a single stage surgery. Below knee splint with foot end elevation on pillow was part of the initial management of simple low energy fractures. We had taken a single incision of average 9cms (range: 5-12cms) in all our patients. The distal tibia fracture reduction was checked under IITV. Utmost care was taken to protect the superficial peroneal nerve. Various reduction techniques were implied to achieve an acceptable reduction. A transverse arthrotomy was done to achieve articular reduction in AO type C fractures (Fig. 1, 2). In comminuted fractures, we used k-wires to hold the reduction. In two cases of intraarticular fractures, we had used lag screws to achieve compression.

Sutures were removed at 14th post-operative day, and radiological follow-up AP and lateral views were taken at three weeks, six weeks, and then at 3,4,6,9 and 12 months. The fracture was confirmed as healed when an obvious callus was seen bridging the fracture ends on both AP and lateral views and also when the patients was able to ambulate on full weight bearing without pain^27^.

Skin incisions, complications related to the soft tissue, wound breakdown and implant exposure were reviewed and recorded for the study. Complications were divided in to major and minor; major complications were those complications that resulted in to morbidities and required further interventions such as deep infections and failure of fixation^10^. Events that did not require any further surgical interventions such as superficial skin infections were considered as minor complications.

## Results

Twenty patients had high energy trauma, five patients had fall from height. We had classified the fractures according to AO classification, 15 patients had extra articular fractures. Five patients had partially articular fractures and five patients had complete articular fractures. Average operation time after trauma was 7.49 days (range: 3-12 days). All the patients were operated as a single procedure where fibula fixation was done first followed by anterolateral plating. We had taken a single incision of average 9cms (range: 5-12cms) in all our patients. Radiological and clinical healing of fracture had occurred at four months in 18 patients, six patients at six months and one patient at eight months. These patients had follow-up for a minimum of one year (range: 12-36 months). Full weight bearing was allowed without any assistance at 5.4 months (range: 3-12 months). We did not have any loss in our follow-up patients. There were no skin and soft tissue healing complications in any of our patients. All these fractures were united at the end of 5.4 months (range: 3-12 months).

In one patient there was a superficial infection that was taken care by regular aseptic dressings and got healed at three weeks without any further deterioration. One patient with uncontrolled diabetes had to undergo debridement and re-closure that got healed at four weeks. Delayed union occurred in two cases. In the first case the fracture got healed by eight months and in another by 11 months. Both of these patients were regular smokers. We did not use any bone grafting for any of our patients. Sensory disturbance on the dorso lateral aspect of foot was observed in two cases. In these patients the movement at the ankle were ranged from 7-14 degrees in dorsi flexion and 5-30 degrees in plantar flexion. Anatomical alignment was within the acceptable range of antero-posterior angulation <10 degrees and the anterolateral angulation of <5 degrees. There was no limb length discrepancy in any of our patients. Ten patients had mild to moderate ankle pain. There was no articular depression in any of our patients. None of the patients required implant removal during the time of this study.

## Discussion

It has been a well-known fact that distal tibia fractures have recently been treated by minimally invasive techniques. Literature says that there is risk of disrupting blood supply with open reduction internal fixation leading to soft tissue healing problems. However, we did not face any of these complications in our patients, infection or wound breakdown with implant exposure^28-29^. Historically, tibial pilon fracture were managed by antero medial approach but one of the major disadvantage in taking this approach is the risk of wound breakdown with exposure of the implant. Implant prominence with antero medial plating has modulated implant removal as a revision surgery. Antero lateral area of distal tibia has shown better soft tissue coverage along with a better direct exposure to the anterolateral fragment. A separate incision for fibula fixation along with conventional distal tibia plating has shown problems with wound healing. Less damage to the periosteal blood supply has been shown in locking plate thus decreasing the incidence of any delayed union or non union or loss of any fixation. Eighteen fractures out of 25 in our study have united within six months with a mean period of 3.95 months (range: 3-6 months)^30^. There are several studies that have reported high complication rates related to soft tissue healing using operative management of tibia fracture^25^.

A study by McFerren *et al* showed the complication rate of 55% that comprise of wound breakdown, deep soft tissue infection, osteomyelitis and superficial wound infection^20^. In order to prevent any soft tissue complication, earlier a 2 stage protocol was recommended that consisted of an initial use of external fixation with or without fibula fixation until the soft tissue envelope recovers sufficiently to allow the definitive fixation^26, 28, 30-33^. In our study we fixed all our fractures in a single stage at a mean time of 8.43 days (range: 4-18 days).

In our surgery we did a stable fixation of fractures with little periosteal damage and minimal soft tissue compromise^34^. We did delay the surgery till the swelling subsided and wrinkles disappeared over the distal tibia. Average delayed time was six days in case of low energy trauma where as in high energy trauma it was 8.43 days (range: 4-18 days). While performing the approach to fibula we tried to limit the amount of dissection over the anterior surface of fibula. In our surgeries since we used the single incision we were not concerned about any skin breech between the two incisions that is usually a concern in conventional approaches. Traditionally a varus injury pattern is advised for medial plating. The anterolateral approach has a greater soft tissue coverage which decreases the trauma to an already compromised soft tissue envelope^11^. We also operated on four tibial pilon fractures that had varus injury through anterolateral plating with a good functional and radiological outcome.

Zackry *et al* postulated that regardless whether the fracture exhibits a varus or valgus pattern, anterolateral plating has identical rigidity from a bio mechanical perspective when compared to medial plating in a varus fracture pattern^35^. Thus, we can confirm that anterolateral plating is a more reliable fixation in a wide category of injuries.

## Conclusion

Distal tibial fractures can successfully be treated by single stage anterolateral plating. Considering a proper surgical timing, respect for soft tissue handling and an incision of average 9cm depending on the needs of fracture pattern a good fixation can be achieved. Moreover, any revision surgery, implant removal due to implant prominence can also be avoided with anterolateral plating.

## Conflict of Interest

The authors declare no conflict of interest.
